# Saffron offers hepatoprotection via up-regulation of hepatic farnesoid-X-activated receptors in a rat model of acetaminophen-induced hepatotoxicity

**DOI:** 10.22038/AJP.2021.18227

**Published:** 2021

**Authors:** Vahid Jamshidi, Seyed Ali Hashemi, Azadeh Khalili, Parviz Fallah, Mohammad Mahdi Ahmadian-Attari, Leila Beikzadeh, Roham Mazloom, Parvaneh Najafizadeh, Gholamreza Bayat

**Affiliations:** 1 *Department of Pharmacology and Toxicology, Faculty of Pharmacy, Pharmaceutical Sciences Branch, Islamic Azad University, Tehran, Iran*; 2 *Department of Pathology, School of Medicine, Alborz University of Medical Sciences, Karaj, Iran*; 3 *Department of Physiology-Pharmacology-Medical Physic, School of Medicine, Alborz University of Medical Sciences, Karaj, Iran *; 4 *Evidence-based Phytotherapy and Complementary Medicine Research Center, Alborz University of Medical Sciences, Karaj, Iran *; 5 *Department of Medical Laboratory Sciences,* *Faculty of Para-Medicine, Alborz University of Medical Sciences, Karaj, Iran*; 6 *Department of Pharmacognosy, Faculty of Pharmacy, Alborz University of Medical Sciences, Karaj, Iran*; 7 *Department of Pharmacology, School of Medicine, Iran University of Medical Sciences, Tehran, Iran*

**Keywords:** Farnesoid X-activated receptor Acetaminophen, Crocus sativus, Crocin, Toxicity

## Abstract

**Objectives:**

The most important toxicity of acetaminophen is hepatotoxicity. Farnesoid X-activated receptors (*FXR*) are one of the nuclear receptor superfamily members which have a pivotal role in the bile acid regulation. The objective of the present study was to examine the role of *FXR* in mediating the hepatoprotective effects of saffron.

**Methods:**

Male Wister rats were randomly allocated into five groups including a control, vehicle, acetaminophen and two saffron extract groups of 150 and 300 mg/kg/day. The liver function and hepatic *FXR* expression were evaluated using biochemical assay and real time RT-PCR, respectively. Data analysis was performed using the one-way ANOVA followed by Duncan's multiple range test.

**Results:**

Levels of aspartate aminotransferase (AST), alanine aminotransferase (ALT), alkaline phosphatase (ALP) and lactate dehydrogenase (LDH) of the acetaminophen group were significantly higher than the control group whereas those of the extract-treated groups were significantly lower than those of the acetaminophen group. The real time RT-PCR findings showed a non-significant down-regulation of *FXR* mRNA expression, however, a dose-dependent *FXR* up-regulation was seen in the groups treated with 150 and 300 mg/kg of the extract for 2.67 (p=0.002) and 10.22 (p=0.0001) fold, respectively.

**Conclusion:**

The main finding of the present study was that the hepatic *FXR* up-regulation had an important role in saffron hepatoprotective activity.

## Introduction

Farnesoid receptors (*FXR*) are members of a nuclear receptor superfamily which have a pivotal role in modulating the bile acid hemostasis and liver disease pathology (Manley and Ding, 2015 ▶). The primary bile acid of chenodeoxycholic acid (CDCA), as the end-product of bile acid synthesis process, is an endogenous ligand the highest affinity for *FXR* (Manley and Ding, 2015 ▶). Regulation of cholesterol synthesis as well as production and/or elimination of bile acids is mediated via these nuclear receptors (Khurana et al., 2011 ▶). As literature shows, there is a direct correlation between the expression of *FXR* and protection against some liver diseases and/or drug-induced hepatotoxicity. There are several lines of evidence which confirm this correlation. Lie et al. showed that hepatic *FXR* expression was reduced in human hepatocellular carcinoma (HCC) compared to normal liver tissue (Liu et al., 2012 ▶). Another clinical study also revealed that the reduction of *FXR* expression in human was associated with development of multiple malignant pathological conditions (Su et al., 2012 ▶). Moreover, an experimental model of knockout *FXR* in aged mice showed higher risk of spontaneously hepatocarcinogenesis (Degirolamo et al., 2015 ▶) Acetaminophen-induced hepatotoxicity in rats was also accompanied with a marked reduction of hepatic *FXR* expression (Adil et al., 2016 ▶). Verbeke et al. findings showed that administration of an *FXR* agonist reduced inflammatory complications in a rat model of toxic cirrhosis (Verbeke et al., 2016 ▶). Also, lipid accumulation in the liver increased in *FXR*-deficient mice under a 1% cholesterol diet (Schmitt et al., 2015 ▶) confirming other aspects of hepatoprotective efficiency of the *FXR* signaling pathway in such hepatic disorders. Besides regulation of bile acid and cholesterol hemostasis, the bile acid-*FXR* interaction participates in modulation of glucose metabolism (Prawitt et al., 2011 ▶; Shapiro et al., 2018 ▶) and balancing inflammatory biomarkers (Shapiro et al., 2018 ▶; Zhu et al., 2016 ▶).

*Crocus sativus* (Saffron) from the Iridaceae family, is well-known in the food industry as a flavoring and coloring agent. Saffron pharmacological benefits are also one of the greatest interests in the pharmaceutical industry. Crocin, crocetin, picrocrocin and safranal are active ingredients of saffron extract (Melnyk et al., 2010 ▶). Several lines of clinical and preclinical evidence are focused on the pharmacological efficacy of crocin and other active ingredients of saffron stigma for therapeutic goals such as blood cholesterol lowering (Mashmoul et al., 2014 ▶), anti-nociceptive (Erfanparast et al., 2015 ▶), anti-inflammatory effects (Yarijani et al., 2017 ▶), neuroprotective (Chen et al., 2015 ▶; Mehri et al., 2012 ▶) and hepatoprotective properties (Hosseini et al., 2015 ▶; Omidi et al., 2014 ▶). The present study was designed to investigate the role of hepatic farnesoid receptors in hepatoprotective activity of saffron hydroalcoholic extract which was quantified in terms of crocin content. The experimental hepatotoxicity was induced using sub-chronic administration of acetaminophen in rats. 

## Materials and Methods

**Chemicals**

Acetaminophen powder was obtained from DarouPakhsh Pharmaceutical Manufacturing Company (Temad Co., Karaj, Iran). Ketamine and Xylazine were purchased from Alfasan (Woerden, Holland).

**Preparing the extract**

Saffron stigma (*Crocus sativus* L.) was purchased from Novin Saffron Co., (Mashhad, Iran) and the hydroalcoholic extract was prepared by percolation method using 80% (v/v). The extract was concentrated using a vacuum rotary evaporator (Hiedolph, Germany) and was left to dry in a desiccator. Yield of extract (w/w) was calculated as weight of dry extract/weight of dry starting material×100. Determination of crocin content of the hydroalcoholic extract of saffron was performed by the quantitative high-performance liquid chromatographic (HPLC) method (Lozano et al., 1999 ▶).

**Animals**

Thirty-five male Wistar rats, weighing 200–250 g, were obtained from Royan Animal Breeding Center, Karaj, Iran. They were kept in the animal house of Alborz University of Medical Sciences, Karaj, Iran under standard conditions with 12 hr light/dark cycle. The temperature and relative humidity were kept at 20–24°C and 50±5%, respectively. During the experiment, animals had free access to food and water. The animal care and experimentation were performed according to the national guidelines and protocols approved by the Research Ethics Committee of Alborz University of Medical sciences in accordance with the National Institute of Health Guide for the Care and Use of Laboratory Animals (NIH Publication No. 85-23, revised 1996). 

**Experimental design and protocol**

Animals were randomly allocated into five groups (n=7 rats each) including a control group, a vehicle group that received carboxymethyl cellulose (CMC) 0.3% as vehicle, an acetaminophen group that received oral acetaminophen 500 mg/kg/day (Venkatesan et al., 2014 ▶) and two groups which simultaneously treated with oral acetaminophen 500 mg/kg/day and saffron extract 150 and 300 mg/kg/day (Amin et al., 2011 ▶). 

Acetaminophen was given by a rat gavage needle at 500 mg/kg/day which was suspended in 0.3% CMC. In the saffron-treated groups, administration of the extract was started 2 hr after acetaminophen administration. The vehicle group received a similar volume of 0.3% CMC. The duration of acetaminophen and saffron extract administration was 28 days. Animals were inspected twice/day for detection of any signs of toxicity and/or mortality. All solutions were prepared freshly and given at the same time on each day. At the end of the experiment period, animals were anesthetized using an intraperitoneal injection of ketamine (60 mg/kg) and xylazine (8 mg/kg). Under deep anesthesia, bilateral thoracotomy was performed and blood samples were obtained gently from the right ventricle and prepared for biochemical analysis. 

**Serum biochemical analysis**

Liver function biomarkers including aspartate aminotransferase (AST), alanine aminotransferase (ALT), alkaline phosphatase (ALP) and lactate dehydrogenase (LDH) levels was measured based on enzymatic reactions by using Pars Azmun commercial kits (Pars Azmun Co, INC, Karaj, Iran) according to the manufacturer’s instructions.

**Histopathological assessments**

The liver was removed and its lateral lobe was cut rapidly and fixed immediately in 10% formalin solution. After dehydration with increasing concentrations of alcohol 70-100%, it was cleared with 100% xylene and embedded in paraffin wax. Prepared tissue blocks were sectioned into a 5-μm thickness on a rotary microtome (DS4802, Didsabz Co.  Urmia, Iran). Hematoxylin and eosin (H&E) staining was performed as a principle method for detecting any pathological signs of toxicity. Specific staining of reticulin was carried out for diagnosis of reticulin fiber deposition. 

**Determination of hepatic** ***FXR*** **gene expression**

*FXR* gene expression analysis was performed by real-time method (Safari et al., 2014 ▶; Safari et al., 2012 ▶). Briefly, about 50 mg of the hepatic tissue was disintegrated using a polytron homogenizer for 1 min (DAIHAN-brand Homogenizing Stirrer, HS-30E; Korea). RNA for evaluating the gene expression was extracted using QIAZOL (Qiagen) based on the manufacturer’s instructions. cDNA synthesis was performed using Reverse Transcriptase cDNA synthesis kit (Fermentas), based on the protocol. Expression of *FXR* was measured by Real-Time PCR using SYBR GREEN (TAKARA). Experiments were performed in duplicates as follows: denaturation at 95°C for 10 min subsequently followed by 45 cycles at 95°C for 10 sec and 60°C for 10 sec and 72°C for 10 sec. The expression level of *FXR* was normalized against *GAPDH* gene. The exact nucleotide sequences of *FXR* and *GAPDH* primers were as follows: *FXR*: forward: TGGGAATGTTGGCTGAATG and reverse: CCTGTGGCATTCTCTGTTTG. *GAPDH*: forward: GCCTTCTCTTGTGACAAAGTG and reverse: CTTCCCATTCTCAGCCTTG. The relative quantification of gene expression was analyzed using REST 2009 

 (V2.0.13).

**Statistical analysis**

The data was analyzed using One-way analysis of variance (ANOVA) and the values are presented as Mean±SEM. Whenever a significant difference was obtained by ANOVA, the source of difference was located using Duncan's 

multiple range test. A *P*-value of less than 0.05 was considered statistically significant. Histological scores were analyzed using Kruskal-Wallis nonparametric test. Data analysis and graphing was performed using Graphpad Prism (V:8.0.2) software. 

## Results

**Yield of extraction and quantification of crocin in the extract**

Total extraction yield (%) was 57.2%. The amount of different types of crocin which was quantified using HPLC is shown in Table 1. As quantitatively shown, crocin 1 was the main crocin type of the hydroalcoholic extract of saffron (17.42%). 

**The effects of acetaminophen on serum markers of liver function**

Table 2 shows the effects of sub-chronic administration of acetaminophen and/or hydroalcoholic extract of saffron on plasma level of liver enzymes. There was no statistically significant difference between the control and vehicle groups regarding ALT, AST, LDH and ALP serum levels. On the other hand, compared to the control group, administration of acetaminophen for 28 days significantly elevated serum levels of AST (p=0.004), ALT (p=0.02) and LDH (p=0.004) enzymes. 

**The effects of saffron extract treatment on serum markers of liver function**

Compared to the acetaminophen group, administration of saffron hydroalcoholic extract at 150 mg/kg/day, significantly reduced the levels of AST (p=0.001), ALT (p=0.01) and LDH (p=0.01).

**Table 1:** Quantitative content of different crocin types in 1 mg/ml saffron stigma extract obtained by HPLC method

**Crocin type**	**RT (min)**	**AUC (%)**	**Concentration (%)**
Crocin 1	26.93	60.39	17.42±1.02
Crocin 2	28.35	31.69	9.11±0.64
Crocin 3	34.05	5.38	2.54±0.30
Crocin 4	39.01	2.53	1.01±0.21

Abbreviations: Retention time (RT), Area under the curve (AUC). The values are reported as AUC (%), RT (min) and concentration (%w/w).

**Table 2:** The effects of acetaminophen and saffron extract treatment on serum concentration of aspartate aminotransferase (AST), alanine aminotransferase (ALT), lactate dehydrogenase (LDH) and alkaline phosphatase (ALP)

	AST	ALT	LDH	ALP
**Control**	106.21±2.49	72.00±2.42	702.66±72.61	822.24±46.87
**Vehicle**	105.83±5.26	70.21±3.27	721.66±31.97	783.24±40.00
**Acetaminophen**	161.20±14.80**	135.65±24.7*	1327.41±158.10**	817.56±56.60
**Saffron-150**	102.40±3.06†††	74.71±4.69††	874.32±61.50††	767.64±47.12
**Saffron-300**	102.74±5.22†††	64.54±4.00†††	693.80±46.32†††●	739.01±29.50

*Significant differences from the control group (*p≤0.05 and **p≤0.01); †significant differences from the acetaminophen-treated group (††p≤0.01 and †††p≤0.001); ●significant differences from the saffron-treated 150 mg/kg/day (●p≤0.05)

The same results were seen following administration of saffron at 300 mg/kg/day as AST (p<0.001), ALT (p<0.001) and LDH (p<0.001) levels were significantly lower than those of the acetaminophen group. Moreover, in extract-treated groups, the reduction in LDH level was dose-dependent (p=0.04). Compared to the control group, the serum levels of ALP did not change due to acetaminophen or saffron extract administration. 

**The effects of acetaminophen on histopathological parameters**

**Figure 1:**
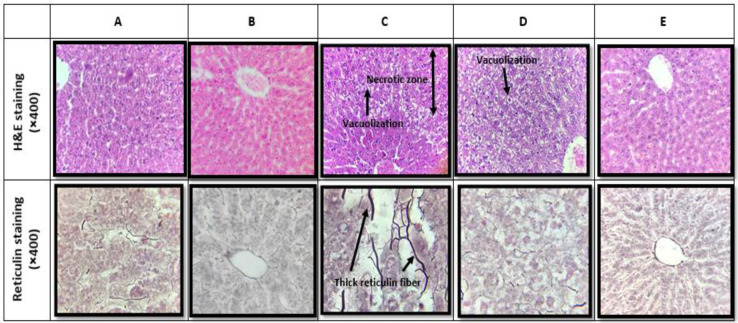
Hematoxylin-eosin (H&E, ×400) and reticulin staining (×400) in (A) control, (B) vehicle-treated, (C) acetaminophen, (D) co-administration of acetaminophen and hydroalcoholic extract of saffron (150 mg/kg/day) and (E) co-administration of acetaminophen and hydroalcoholic extract of saffron (300 mg/kg/day). Reticulin fiber deposition was markedly developed due to acetaminophen treatment (C) which was restored after co-treatment with the saffron extract (D and E)

The section samples of H&E and reticulin staining, as well as the scoring values of liver pathological alteration are shown in Figure 1 and Table 3, respectively. As shown in Figure 1A and B, in the vehicle-treated group, H&E and reticulin staining did not show any histopathological alteration compared to the control ones.

In contrast, the acetaminophen-treated group was associated with marked alteration of liver histology (Figure 1C) such as marked glycogen depletion, congestion, sinusoidal dilation, vacuolization, bile stasis, Kupffer cell hyperplasia and necrosis (Table 3). Moreover, reticulin staining revealed thick reticulin fibers in acetaminophen liver slide (Table 3).

**The effects of saffron extract treatment on histopathological parameters**

As shown in Figures 1D and E and Table 3, some pathological damages including congestion, sinusoidal dilation, vacuolization, bile stasis and plugs as well as necrosis were markedly restored following saffron extract administration. As determined by reticulin staining and in comparison with the control one, a significant reduction in reticulin fiber deposition was seen in both saffron extract-treated groups.

**The effects of acetaminophen on the expression of hepatic farnesoid receptors**

**Figure 2:**
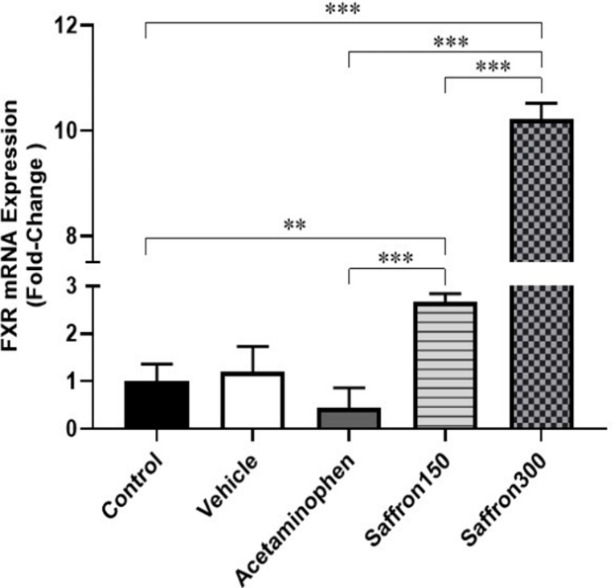
Fold change expression of hepatic *FXR* in (A) control, (B) vehicle-treated, (C) acetaminophen-treated (D) co-administration of acetaminophen and hydroalcoholic extract of saffron (150 mg/kg/day), and (E) co-administration of acetaminophen and hydroalcoholic extract of saffron (at 300 mg/kg/day)

The results of real time RT-PCR showed that vehicle treatment, compared to control, did not have any significant effect on the expression of hepatic *FXR* receptors. Compared to the control group, administration of acetaminophen was associated with a 0.44-fold reduction in hepatic mRNA expression, however it was not statistically significant (Figure 2).

**Table 3:** Pathological alteration of the liver in H&E and reticulin staining

**Number**	**Pathological signs**	**Control**	**Vehicle**	**Acetaminophen**	**Saffron 150**	**Saffron 300**
** H&E staining**						
**1**	Glycogen depletion	0	0	1*	0	0
**2**	Congestion	0	0	3***	1	1
**3**	Sinusoidal dilation	0	0	2***	1	1
**4**	Inflammatory infiltration (Lymphocytic infiltration)	0	0	2	1	0
**5**	Vacuolization	0	0	3***	1*	0
**6**	Bile stasis	0	0	2*	0	0
**7**	Bile plugs	0	0	1	0	0
**8**	Kuffer cell hyperplasia	0	0	2*	2*	3***
**9**	Pyknosis	0	0	1	0	0
**10**	Necrosis	0	0	2*	0	0
**Reticulin staining**						
**Reticulin fibers**		0	0	2***	0###	0###

Histological changes were scored as none (0), active damage less than 25% (+), active damage less than 50% (++), active damage less than 75% (+++) damage and active. *Significant differences from the control group (*p≤0.05, **p≤0.01 and ***p≤0.001); #significant differences from the acetaminophen-treated group (###p≤0.001).

**The effects of saffron extract treatment on the expression of hepatic farnesoid receptors**

The findings also showed a significant up-regulation of hepatic *FXR* following co-administration of saffron extract and acetaminophen. As shown in Figure 2, compared to the control group, the expression of hepatic *FXR* was 2.67- (p=0.002) and 10.22-fold (p<0.001) higher in the groups treated with saffron 150 and 300 mg/kg/day, respectively. In comparison to the acetaminophen group, saffron 150 and 300 mg/kg/day significantly increased hepatic expression of *FXR* to 6.00 (p<0.001) and 22.94 (p<0.001) times, respectively. The observed up-regulation of the receptor by saffron extract had a dose-dependent behavior as the *FXR* mRNA expression in the higher dose group was 3.82 (p<0.001) times higher than the lower dose group.

## Discussion

The main objective of the present study was to evaluate the role of the farnesoid-X activated receptors in hepatoprotective effects of saffron extract. Our findings indicated that co-administration of acetaminophen with saffron extract was associated with a significant up-regulation of hepatic farnesoid-X activated receptors. The observed up-regulation of the receptor was clearly dose-dependent. 

Saffron, has several pharmacological activity including cardioprotective (Mehdizadeh et al., 2013 ▶), neuroprotective and memory-improving (Purushothuman et al., 2013 ▶), hepatoprotective (Hosseini et al., 2015 ▶), cytoprotective activity against some cytotoxic agents such as chemotherapy drugs (D'Alessandro et al., 2013 ▶; Jnaneshwari et al., 2013 ▶), as well as anticancer properties (D'Alessandro et al., 2013 ▶). The biological and pharmacological activity of saffron extract has been attributed to its main active ingredients such as crocin, crocetin, picrocrocin and safranal. Crocin, is the main constituent which is involved in most of the pharmacological activity of saffron extract (Melnyk et al., 2010 ▶). As literature data shows it has a drastic radical scavenging and therefore antioxidative activity (Dar et al., 2017; Jnaneshwari et al., 2013 ▶). According to previous studies, there is a positive correlation between crocin content and hepatoprotective activity of saffron extract. As a hepatoprotective agent, most of the experiments have focused on anti-inflammatory (Moossavi et al., 2016 ▶; Yarijani et al., 2017 ▶) and antioxidant properties (Khorasany and Hosseinzadeh, 2016 ▶; Moossavi et al., 2016 ▶) as the main mechanism of saffron effect. But according to its broad range of activity, it seems that it is more than just an antioxidant agent. So, the present study focused on the farnesoid receptors as another crocin target. According to our results, co-administration of saffron extract significantly restored hepatic function and histology via up-regulation of *FXR*s. Restoration of the liver function markers was in consistence with other experimental studies (Aml F. Elgazar et al., 2013 ▶; Mashmoul et al., 2016 ▶). In contrast, a recent randomized clinical trial study did not find any significant effects for *C. sativus* on liver enzymes in type 2 diabetic patients (Ebrahimi et al., 2019 ▶). The existence of thick visible reticulin fiber due to acetaminophen treatment, revealed that sub-chronic oral administration of acetaminophen was associated with marked necrosis. The observed findings were in parallel with the enzyme abnormality in this group. In the extract-treated groups also the findings of biochemical assay were in consistence with histopathological assessment. In this regard, following administration of saffron extract, hepatic architecture showed a significant improvement. 

In the present study, the protective effect of saffron at the higher dose (300 mg/kg/day) was more pronounced as the observed up-regulation was more than 3 times (3.82) compared to that of the lower dose (150 mg/kg/day). The role of *FXR* in the hepatoprotective activity of other herbal remedies has been also experimentally investigated for chicory and silymarin extract (Khalili et al., 2021 ▶). The *FXR* is a member of a nuclear receptor superfamily that is physiologically activated by endogenous bile acids. Several lines of evidence indicate that activation of *FXR* receptors has a pivotal role in preventing hepatic abnormalities. As previous findings show, hepatic expression of *FXR* is impaired during some liver pathological conditions such as hepatocellular carcinoma (Su et al., 2012 ▶), alcoholic- cholestasis (Manley and Ding, 2015 ▶) and nonalcoholic steatohepatitis (Armstrong and Guo, 2017 ▶). Increased mRNA level of inducible nitric oxide synthase (iNOS) and cyclooxygenase-2 (COX-2), in *FXR*- deficient mice is an important indicator of the protective role of hepatic *FXR* against inflammatory reactions (Wang et al., 2008 ▶). In another study, Adil et al. showed that acetaminophen-induced hepatotoxicity (700 mg/kg) was also associated with a marked reduction of hepatic *FXR* gene expression in rats (Adil et al., 2016 ▶). In contrast, in the present study, acetaminophen (500 mg/kg)-induced down-regulation of *FXR* mRNA was not significant. Similar result has been also reported in our other previous study (Khalili et al., 2021 ▶). It seems that the higher dose of acetaminophen was required for induction of a significant *FXR* gene down-regulation. 

On the other hand, there are findings which show that administration of bile acids and/or *FXR* synthetic ligands improve such pathological conditions (Abenavoli et al., 2018 ▶). According to Zhang et al. study, administration of an *FXR* agonist, WAY-362450, was associated with a marked reduction of inflammatory cell infiltration in a mouse model of non-alcoholic steatohepatitis (Zhang et al., 2009 ▶). The mentioned effect was not seen in *FXR*-deficient mice (Zhang et al., 2009 ▶). Moreover, pretreatment of HepG2 cells and mouse primary hepatocytes with another *FXR* agonist, led to suppression of nuclear factor kappa (NF-κB), an inflammatory nuclear factor, gene expression (Wang et al., 2008 ▶). Our findings are in agreement with that of the previous ones so the up-regulation of hepatic *FXR* was associated with functional and histopathological improvement of the liver. So, such a significant up-regulation of *FXR* relative gene expression, suggests that the hepatoprotective activity of saffron extract might be directly or indirectly mediated via interactions with the *FXR* signaling pathway. Whether crocin and/or other active constituents of saffron did have a direct agonistic or allosteric modulator activity on *FXR* is not clear. Also, whether or not other mechanisms are involved in saffron hepatoprotective activity, remains unclear. 

The main limitation of the present study was neglecting the interaction of farnesoid-glutathione pathways. Toxic doses of acetaminophen lead to glutathione storage depletion which triggers hepatotoxicity. Investigating any changes in glutathione levels and the genes involved in this pathway, along with alterations in the levels of the nuclear receptors, can provide valuable information.

The present findings suggest that hepatic *FXR* signaling pathway has an important role in the hepatoprotective activity of saffron extract. In this regard, receptor up-regulation following extract administration exhibits a dose-dependent behavior. Further investigation is required to elucidate the exact mechanism of crocin-*FXR* interaction. 

## Conflicts of interest

The authors have declared that there is no conflict of interest. 
